# Hyperemesis gravidarum and risk of cancer in offspring, a Scandinavian registry-based nested case–control study

**DOI:** 10.1186/s12885-015-1425-4

**Published:** 2015-05-13

**Authors:** Kathrine F. Vandraas, Åse V. Vikanes, Nathalie C. Støer, Rebecca Troisi, Olof Stephansson, Henrik T. Sørensen, Siri Vangen, Per Magnus, Andrej M. Grjibovski, Tom Grotmol

**Affiliations:** 1 Department of Genes and Environment, Norwegian Institute of Public Health, PO Box 4404, Nydalen 0403 Oslo, Norway; 2 Norwegian National Advisory Unit on Women’s Health, Oslo University Hospital, PO box 4950, Nydalen Oslo, Norway; 3 Department of Medical Epidemiology and Biostatistics, Karolinska Institutet, Stockholm, Sweden; 4 Divisions of Cancer Epidemiology and Genetics, National Cancer Institute, National Institutes of Health, Department of Health and Human Services, 9000 Rockville Pike, Bethesda, MD 20892 USA; 5 Clinical Epidemiology Unit, Department of Medicine, Karolinska University Hospital and Institute, SE-141 86 Stockholm, Sweden; 6 Department of Women and Children’s Health, Division of Obstetrics and Gynecology Karolinska University Hospital and Institute, SE-141 86 Stockholm, Sweden; 7 Department of Clinical Epidemiology, Aarhus University Hospital, 44 Norrebrogade, 8000 Aarhus, Denmark; 8 Department of International Public Health, Norwegian Institute of Public Health, PO Box 4404, Nydalen 0403 Oslo, Norway; 9 International School of Public Health, Northern State Medical University, Troitsky av.51, Arkhangelsk, Russia 163000; 10 Department of Preventive Medicine, International Kazakh-Turkish University, Esimkhan str.2, Turkestan, Kazakhstan; 11 Cancer Registry of Norway, PO Box 5313, Majorstuen N-0304 Oslo, Norway; 12 The Intervention Center, Oslo University Hospital, PO Box 4950, Nydalen Oslo, Norway

**Keywords:** Hyperemesis, Cancer, Fetal programming

## Abstract

**Background:**

Hyperemesis gravidarum is a serious condition affecting 0.8–2.3 % of pregnant women and can be regarded as a restricted period of famine. Research concerning potential long-term consequences of the condition for the offspring, is limited, but lack of nutrition *in-utero* has been associated with chronic disease in adulthood, including some cancers. There is growing evidence that several forms of cancer may originate during fetal life. We conducted a large study linking the high-quality population-based medical birth- and cancer registries in Norway, Sweden and Denmark, to explore whether hyperemesis is associated with increased cancer risk in offspring.

**Methods:**

A registry-based nested case–control study. Twelve types of childhood cancer were selected; leukemia, lymphoma, cancer of the central nervous system, testis, bone, ovary, breast, adrenal and thyroid gland, nephroblastoma, hepatoblastoma and retinoblastoma. Conditional logistic regression models were applied to study associations between hyperemesis and risk of childhood cancer, both all types combined and separately. Cancer types with five or more exposed cases were stratified by age at diagnosis. All analysis were adjusted for maternal age, ethnicity and smoking, in addition to the offspring’s Apgar score, placental weight and birth weight. Relative risks with 95 % confidence intervals were calculated.

**Results:**

In total 14,805 cases and approximately ten controls matched on time, country of birth, sex and year of birth per case (147,709) were identified. None of the cancer types, analyzed combined or separately, revealed significant association with hyperemesis. When stratified according to age at diagnosis, we observed a RR 2.13 for lymphoma among adolescents aged 11–20 years ((95 % CI 1.14–3.99), after adjustment for maternal ethnicity and maternal age, RR 2.08 (95 % CI 1.11–3.90)). The finding was not apparent when a stricter level of statistical significance was applied.

**Conclusions:**

The main finding of this paper is that hyperemesis does not seem to increase cancer risk in offspring. The positive association to lymphoma may be by chance and needs confirmation.

## Background

Hyperemesis gravidarum is characterized by severe nausea and vomiting during early pregnancy resulting in maternal weight loss, nutritional deficiencies and hospital admissions [1]. Little is known of the underlying causes and consequences of the condition. Genetic, hormonal as well as environmental factors are believed to play important roles [2]. Previous research has primarily focused on short-term outcomes associated with hyperemesis, with inconsistent associations demonstrated for preterm birth, low birth weight and risk of offspring small for gestational age [3]. Two recent, large studies based on Norwegian registry data, demonstrated no clinically significant impact of hyperemesis on birth outcomes [4-6]. However, individual studies have reported that hyperemesis may have a long-term impact on disease patterns later in life, including increased risk of hypertension and reduced insulin sensitivity [4,5]. Furthermore, The United Kingdom Childhood Cancer Study (UKCCS) found a 3.5-fold increase in risk for all forms of leukemia among offspring of mothers with severe hyperemesis [6], and an American study reported that hyperemesis was associated with a four-fold increase in testicular cancer risk among male offspring [7].

The fetal programming hypothesis suggests that adverse exposures during critical periods of embryonic development, in particular the first trimester, may permanently alter disease-susceptibility in later life [8]. Lack of nutrition is identified as key negative stimulus, which may cause changes in the fetal circulation, prioritizing essential growth (brain sparing) at the expense of other organs and tissues, or in the epigenome of the fetus. These adaptive mechanisms may have long-term impact on the functioning of these organs and biological systems, resulting in increased susceptibility to diseases in adulthood. For example, several studies have demonstrated that maternal starvation increases the risk of non-communicable diseases in adulthood of the offspring, such as hypertension, glucose-intolerance, coronary heart disease and some forms of cancer [9-11]. These long-term effects of exposure to starvation in fetal life are irrespective of birth weight [11], which suggests that even short-term nutritional deprivation is important. Although relatively rare, the incidence of cancer among children and adolescents is increasing and is in many countries the leading cause of disease-related death in this age-group [12]. Only a small percentage of these cancers are caused by an inherited genetic mutation, suggesting that cancer risk in this group is under influence of many modifiable risk factors. These factors may act through epigenetic pathways during fetal development [13].

Hyperemesis is a severe complication occurring in early pregnancy that in many ways mimics starvation thereby providing a model to explore the consequences of under nutrition during a critical period of fetal development. Specific hormonal alterations related to hyperemesis may also influence epigenetic mechanisms affecting the offspring’s susceptibility to other diseases, such as cancer. Given the sparse data on associations between maternal hyperemesis and cancer risk in offspring, large, population-based studies based on data collected in a standardized protocol are needed. The aim of this study was therefore to investigate whether hyperemesis is associated with cancer in the offspring, using merged national medical birth- and cancer registries in Norway, Sweden and Denmark.

## Methods

This nested case–control study is based on pooled data from population-based registries in each of the Scandinavian countries. The unique identification number assigned to all citizens in these countries at birth or upon immigration was used to link the medical birth registries (MBRs) to the national cancer registries. The MBRs in Norway, Sweden and Denmark, founded in 1967, 1973 and 1977, respectively, are based on mandatory reporting of all births on standardized forms, completed by the attending midwife or physician shortly after birth and supplemented by the antenatal health card and hospital records. The MBRs contain information on maternal background, pregnancy and birth, and selected short-term outcomes for the offspring. The Scandinavian cancer registries, established in 1943 (Denmark), 1951 (Norway) and 1957 (Sweden), are also population-based, with mandatory reporting of all incident tumors. Data in these registries have been reported to be complete and of high quality [14-18].

For the Norwegian and Swedish data, information on maternal country of birth was obtained from Statistics Norway and Statistics Sweden, respectively. In Denmark, demographic variables were obtained from the Civil Registration System. Information on smoking habits became available in Sweden in 1982, in Denmark in 1991 and in Norway in 1999. For Apgar scores, information was available in Sweden in 1972, in Denmark in 1978 and in Norway in 1976. Placental weight was available in Sweden during 1982–1999, in Denmark in 1997 and in Norway in 1999. Because these data became available at different times in the three countries, the number of missing values is relatively high in our study.

Our study included the twelve most common types of cancer in childhood and adolescence, defined according to the 10^th^ edition of the International Classification of Disease (ICD-10); leukemias (C91-95), lymphomas (C81-C85), tumors of the brain and nervous system (C70-72 and D42-43), breast, females only (C50), bone (C40-C41), testis (C62), ovary (C56), thyroid gland (C73), adrenal gland (C74), retinoblastoma (C69.2), Wilms’ tumor (C64.9) and hepatoblastoma (C22). Cases were Scandinavian children and adolescents registered in the MBRs at birth, diagnosed with one of the above types of cancer before the age of 21 years and registered in the corresponding National Cancer Registry. The first 21 years of life were selected to focus primarily on the potential effect of perinatal exposure. Only singletons born between 23–43 weeks of gestation, and only primary cases of cancers were included.

For each case, we sampled up to ten controls who were cancer-free at time of diagnosis for the case, and matched by birth registry, sex and year of birth. Children with Downs’s syndrome were excluded as they are known to be at higher risk for several types of cancer.

In Sweden, hyperemesis was defined through ICD-8 codes 638.0 and 638.9 until 1987, ICD-9 code 643 until 1997 and subsequently with ICD-10 code O21, O21.1 and O21.9, gathered from the MBR and supplemented from the National Patient Registry (NPR) to increase the validity of the diagnosis. In Norway and Denmark, hyperemesis was defined through ICD-8 codes until ICD-10 codes were available. In Denmark, information on hyperemesis was gathered from the NPR, while in Norway this information was obtained from the MBR solely.

Maternal country of birth, smoking (smoker/nonsmoker) and age (in five-year age-groups) were considered as possible confounders, and adjusted for, as were placental weight (less than 500, 500–999 and equal to or heavier than 1000 g and missing), birth weight (less than 1500, 1500–2499, 2500–3499, 3500–3999, 4000–4499 and birth weights equal to or above 4500 g and missing) and Apgar score (at one and five mins; equal to or below seven or higher than seven and missing). In line with previous research on hyperemesis, maternal country of birth was categorized into six immigrant groups that were culturally and geographically related.

Conditional logistic regression models were used to study associations between hyperemesis and all selected types of childhood cancer. The models were stratified by age for each cancer type with five or more exposed cases. The regression models were adjusted by maternal age, ethnicity and smoking, in addition to offspring’s Apgar score, placental weight and birth weight. We then implemented a backward elimination procedure, removing explanatory variables one by one and observing if the estimate changed. Crude and adjusted relative risks (RR) were calculated with 95 % confidence intervals (CI). Due to multiple testing, we performed the analyses with 99 % confidence intervals as well, for selected cancer types. SPSS for Windows version 20.0 (SPSS Inc., Chicago, IL, USA) was used for all analyses.

The study was approved by the Danish Data Protection Agency (record no 2008-41-2767) and the Regional Ethical Board in Stockholm, Sweden and the Regional Ethical Committee in Oslo, Norway.

## Results

Demographic variables for mothers and offspring are presented in Table 1. Ninety-seven (0.7 %) cases were exposed to hyperemesis during pregnancy. A high percentage of missing values was observed for maternal smoking, placental weight and Apgar score after one and five mins: 77.4 %, 80.0 % and 16.3/14.1 %, respectively. Neither maternal nor fetal variables differed substantially between cases and controls.

**Table 1:** Demographic characteristics of all mothers, mothers of cases and mothers of controls, and birth outcomes for all offspring, for cases and controls

	Cases (%)	Controls (%)	Total
Maternal country of birth			
Europe, USA, Canada	14.102 (95.3)	139.949 (94.7)	154,051
Middle-East*	262 (1.8)	2.732 (1.8)	2.994
Africa excluding North-Africa	77 (0.5)	823 (0.6)	900
Asia**	155 (1.0)	1.897 (1.3)	2.052
Central and South-America	105 (0.7)	1.085 (0.7)	1.190
Other countries and missing	104 (0.7)	1.223 (0.8)	1.327
Maternal age, in years			
<20	601 (4.1)	6.227 (4.2)	6.828
20–24	3.423 (23.1)	35.446 (24.0)	38.869
25–29	5.385 (36.4)	53.690 (36.3)	59.075
30–34	3.716 (25.1)	36.561 (24.8)	40.277
>34	1.680 (11.3)	15.785 (10.7)	17.465
Smoking***			
Nonsmoker	2.042 (13.8)	21.011 (14.2)	23.053
Smoker	1.264 (8.5)	12.418 (8.4)	13.682
Missing	11.499 (77.7)	114.280 (77.4)	125.779
Hyperemesis status			
HG +	97 (0.7)	818 (0.6)	915
HG -	14.708 (99.3)	146.891 (99.4)	161.599
Birth weight (gr)			
<1500	97 (0.7)	1.021 (0.7)	1.118
1500–2499	549 (3.7)	5.602 (3.8)	6.151
2500–3499	6.046 (40.8)	63.972 (43.3)	70.018
>3500	8.067 (54.5)	76.771 (52.0)	84.838
Missing	46 (0.3)	343 (0.2)	389
Placental weight (gr)****			
<500	411 (2.8)	4.177 (2.8)	4.588
500–999	2.492 (16.8)	24.872 (16.8)	27.364
≥1000	112 (0.8)	929 (0.8)	1.041
Missing	11.790 (79.6)	117.731 (79.7)	129.521
Apgar score after 1 min*****			
< 7	659 (4.5)	5.597 (3.8)	6.256
≥ 7	11.760 (79.4)	117.986 (79.9)	129.746
Missing	2.386 (16.1)	24.126 (16.3)	26.512
Apgar score after 5 min*****			
< 7	161 (1.1)	1.400 (0.9)	1.561
≥ 7	12.558 (84.8)	125.480 (85.0)	138.038
Missing	2.086 (14.1)	20.829 (14.1)	22.915
Total	14.805	147.709	162.514

*Middle-East includes Turkey, Lebanon, Syria, Palestine, Iraq, Morocco, Algeria, Tunisia, Libya, Egypt, Iran

**Asia includes Pakistan, India, Sri-Lanka, Vietnam, Thailand, Philippines, China, South-Korea, Japan

***Available from 1991 in Denmark, 1999 in Norway and 1982 in Sweden

****Available from 1997 in Denmark, 1999 in Norway and in for the years 1982–1999 in Sweden

*****Available from 1991 in Denmark, 1999 in Norway and 1982 in Sweden

Numbers in parentheses indicate percentage distributions within the categories of each variable among all offspring, among cases and among controls

Leukemia was the most common type of childhood cancer, comprising almost 35 % of the cases (*n* = 5114), followed by tumors of the central nervous system (30.6 %) and lymphoma (12.5 %). Cancers of the breast, testis, thyroid and ovary were more common in the oldest age group, while tumors of the adrenal gland, nephroblastoma and retinoblastoma were more frequent among the youngest age-groups (Table 2). Leukemia was most common under 11 years of age, while lymphoma peaked among adolescents and young adults. In total, 63 % of cases were between 0 and 10 years old at time of diagnosis.

**Table 2:** Number of cases and age at diagnosis according to cancer type. Numbers in parentheses indicate percentage distributions among age categories for each cancer type

Type of cancer	Age at diagnosis		
	0–10	11–20	N
Leukemia	4.114 (80.4)	1.000 (19.6)	5.114
Central nervous system	3.003 (66.4)	1.521 (33.6)	4.524
Lymphoma	491 (26.5)	1.362 (73.5)	1.853
Testis	154 (18.2)	693 (81.8)	847
Nephroblastoma	422 (95.7)	19 (4.3)	441
Adrenal gland	386 (92.6)	31 (7.4)	417
Primary bone	163 (41.4)	231 (58.6)	394
Retinoblastoma	344 (99.7)	1 (0.3)	345
Thyroid gland	35 (10.2)	308 (89.8)	343
Ovary	67 (22.6)	229 (77.4)	296
Hepatoblastoma	172 (80.8)	41 (19.2)	213
Breast	1 (5.6)	17 (94.4)	18
Total	9.352 (63.2)	5.453 (36.8)	14.805

No association between hyperemesis and childhood cancer was observed for all selected cancer types combined (Table 3). This was unchanged after adjustment for maternal age and country of birth. For cancers of the breast, bone, testis, ovary, thyroid and adrenal gland, in addition to retinoblastoma and hepatoblastoma, fewer than five cases each had been exposed to hyperemesis. When the model was stratified according to age at diagnosis for the remaining cancer types (Table 4), a significant association between hyperemesis and lymphoma was observed in offspring aged 10–20 years (RR = 2.13 (95 % CI: 1.14–3.99)), which was not observed in the younger age-group. Adjustment for potential confounders did not significantly change the estimate (RR = 2.08 (95 % CI: 1.11–3.90)). None of the other selected cancer types were significantly associated with maternal hyperemesis. When applying an alpha-level of 0.01, the risk of lymphoma in the highest age-group was no longer statistically significant; RR 2.13 (99 % CI: 0.93–4.85) and aRR 2.08 (99 % CI: 0.91–4.45) (results not shown in table).

**Table 3:** Relative risk (RR) of cancer in offspring according to maternal hyperemesis gravidarum (HG)- status for selected types of cancer combined and separately, with 95 % confidence intervals (95 % CI)

	N	Cases	Controls	Crude RR (95 % CI)	Adjusted RR* (95 % CI)
Total	14.805	HG+ 97	HG+ 818	1.18 (0.95-1.46)	1.19 (0.97-1.48)
		HG- 14.708	HG- 146.891		
Leukemia	5.114	HG+ 28	HG+ 280	0.99 (0.67-1.47)	1.00 (0.68-1.48)
		HG-5.086	HG- 50.845		
Central nervous	4.524	HG+ 29	HG+ 242	1.20 (0.81-1.78)	1.24 (0.84-1.83)
system		HG- 4.495	HG- 45.026		
Lymphoma	1.853	HG+ 15	HG+ 102	1.70 (0.98-2.96)	1.68 (0.97-2.92)
		HG- 1.838	HG- 19.973		
Testis	847	HG+ 4	HG+ 51	0.87 (0.31-2.43)	0.87 (0.31-2.43)
		HG- 843	HG- 9.190		
Nephroblastoma	441	HG+ 8	HG+ 48	1.97 (0.91-4.23)	2.01 (0.93-4.34)
		HG- 433	HG- 4.850		
Adrenal gland	417	HG+ 3	HG+ 28	1.12 (0.34-3.75)	1.14 (0.34-3.80)
		HG- 414	HG- 4.567		
Primary bone	394	HG+ 2	HG+ 15	1.40 (0.31-6.20)	1.38 (0.31-6.15)
		HG-392	HG- 4.286		
Retinoblastoma	345	HG+ 3	HG+ 36	0.97 (0.29-3.20)	0.97 (0.29-3.20)
		HG- 342	HG- 3.725		
Thyroid gland	343	HG+ 4	HG+ 21	2.11 (0.71-6.29)	2.21 (0.73-6.71)
		HG- 339	HG- 3.836		
Ovary	296	HG+ 1	HG+ 20	0.50 (0.07-3.72)	0.48 (0.06-3.62)
		HG- 295	HG- 3.193		
Hepatoblastoma	213	HG+ 0	HG+ 13	-	-
		HG- 213	HG- 2.340		
Breast	18	HG+ 0	HG+ 2	-	-
		HG- 18	HG- 187		

*Adjusted for maternal age and maternal country of birth

**Table 4:** Relative risk (RR) of cancer in offspring according to maternal hyperemesis gravidarum (HG)- status for selected types of cancer combined and separately for the most common types according to age at diagnosis, with 95 % confidence intervals (95 % CI)

Cancer type	Age at diagnosis, in years	Crude RR (95 % CI)	Adjusted RR* (95 % CI)*
All selected cancer forms			
n = 9.352	0–10	1.16 (0.90–1.51)	1.18 (0.91–1.53)
n = 5.453	11–20	1.21 (0.84–1.74)	1.21 (0.84–1.74)
Leukemia			
n = 4.114	0–10	1.07 (0.70–1.64)	1.08 (0.71–1.65)
n = 1.000	11–20	0.70 (0.25–1.93)	0.70 (0.25–1.93)
Central nervous system			
n = 3.003	0–10	1.18 (0.74–1.89)	1.22 (0.76–1.94)
n = 1.521	11–20	1.27 (0.63–2.56)	1.27 (0.62–2.56)
Lymphoma			
n = 491	0–10	0.95 (0.29–3.12)	0.94 (0.28–3.10)
n = 1.362	11–20	2.13 (1.14–3.99)	2.08 (1.11–3.90)
Nephroblastoma			
n = 422	0–10	2.02 (0.94–4.35)	2.06 (0.95–4.45)
n = 19	11–20	–	–

*Adjusted for maternal age and maternal country of birth

## Discussion

In this study, the main results displayed no association between hyperemesis and cancer risk in offspring. This is reassuring news for women suffering from hyperemesis, which is the most common cause of hospital admissions in early pregnancy. However, in the age group 10–20 years we observed a significant positive association between hyperemesis and lymphoma. As we performed multiple analyses, we explored the association with stricter criteria for statistical significance. Our main finding was not significant at an alpha-level of 0.01. Further investigation on the impact of offspring’s age, revealed that only the oldest adolescents in the highest age-group were at increased risk. The potential effect of adverse perinatal exposure becomes more difficult to isolate from later environmental influences with increasing age of the offspring. This warrants caution in the interpretation of the findings. However, according to the hypothesis of fetal programming, adverse exposure *in-utero* may increase an individual’s vulnerability for disease in adulthood, co-acting with environmental exposures. Despite the pooling of data from Scandinavia, the numbers of cases exposed to hyperemesis was still low, limiting the ability to detect significant associations. Stratifying by birth registry, the same positive tendency regarding lymphoma risk was observed both in Sweden and Norway. In Denmark there were not enough cases to perform the analyses. Since current knowledge on the long-term consequences of hyperemesis for the offspring is limited, these findings warrant further research on the topic.

There is increasing evidence that several sub-types of hematological malignancies can originate *in-utero* [19,20]. Single studies have also reported increased risk of cancer in offspring following hyperemesis exposure. In the United Kingdom Childhood Cancer Study (UKCCS), a positive association was reported between severe hyperemesis and acute lymphatic leukemia and acute myeloid leukemia with an OR of 3.6 (95 % CI: 1.3-10.1). For non-Hodgkin’s lymphoma an OR of 6.8 was reported, but the association did not reach the level of statistical significance [21]. The UKCCS was based on high-quality data and specifically designed to explore perinatal risk factors for childhood cancer. However, the number of exposed cases was low, with only eight cases in total. Based on 28 exposed leukemia cases, we did not observe any such association.

How hyperemesis may increase the risk of lymphoma is a matter of speculation. Lymphoma has been linked to fetal growth and low birth weight [22]. The association of hyperemesis with low birth weight has been inconsistent [3,5,6], possibly because the maternal hunger-period is short, causing any weight loss early in pregnancy to be compensated for in the remaining weeks. Also, efficient treatment may secure fetal growth. However, the general environment in utero could still be adversely affected [11]. Previous studies exploring the effect of famine exposure confined to early pregnancy have reported negative outcomes for long-term health regardless of birth weight [11,23,24].

The underlying biology behind the programming of cancer susceptibility *in-utero* is unknown but is likely to involve epigenetic mechanisms. Epigenetics refer to any change to the genome which does not include alterations in the nucleotide sequence. DNA methylation and histone modification are two important epigenetic mechanisms by which the gene expression may be modified [25]. The epigenetic changes may affect different regulatory pathways such as the production of stem cells or hormones, which may alter organogenesis [13]. DNA methylations have been observed in several steps of carcinogenesis [26]. It has also been suggested that nutritional restriction may cause changes to the fetal blood circulation, sparing the brain at the expense of other organs and tissues during a “window of vulnerability” in fetal development. Altered perfusion patterns may result in long-term increased disease susceptibility.

Although the etiology of hyperemesis is unknown, several studies have suggested that elevated levels of estrogen and human chorionic gonadotropin (hCG) are important risk factors [27,28]. hCG can act as a growth factor and is associated with placental- and germ cell-cancers in particular, but subtypes of the molecule are believed to be produced in most advanced malignancies [29,30]. During pregnancy, estrogen levels are more than ten times higher than normal, and can be even higher among women with hyperemesis. Pregnancies with a female or multiple fetuses both have been associated with higher levels of estrogen as well as higher risk of hyperemesis [27,31,32]. Estrogens may be oncogenic to hematopoietic cells, and some studies have shown an association between estrogen exposure and leukemia [33]. It is not known whether the same association exists between hyperemesis and lymphoma.

As risk of breast cancer has been linked to estrogen exposure *in-utero* [34], offspring born to hyperemetic mothers may also be at increased risk. While we did not find such an association, we only followed offspring to age 21. Given that breast cancer has a median - age of incidence of about 60 years in western countries [35], our dataset was not appropriate for studying a possible association between *in-utero* exposure to hyperemesis and subsequent breast cancer. At the same time, with age it becomes more difficult to distinguish the biological from the environmental impacts on cancer risk. In addition, as the MBRs were founded in the 1960s, the majority of offspring have yet to enter higher risk age-group.

In contrast to findings of an American study of 131 men with testicular cancer, we found no association between hyperemesis and risk of testicular cancer in offspring [36]. However, this study dating back to 1979, was small and included only eight cases of testicular cancer following a hyperemetic pregnancy. To our knowledge, such an association has not been reported since. Testicular cancer in childhood is rare and differs histologically, genetically and etiologically from that observed in adolescence and adulthood [37]. The condition has been associated with both a high ponderal index and high birth weight, suggesting links between childhood testicular cancer, the intrauterine environment and fetal growth. As in the case of breast cancer, inclusion of older age-groups of cases might have yielded interesting findings. Still, it would have been difficult to isolate the effect of the intrauterine environment on risk of these cancers.

The major strength of this study is its large sample size, resulting from collaboration between the three Scandinavian countries. As well, merging population-based registries provided a relatively high number of cases making selection bias therefore unlikely and increasing the generalizability of our results. Registration of hyperemesis was performed prior to development of cancer in the offspring, which eliminated the risk of recall-bias. The MBRs offer extensive information on maternal and fetal variables making it possible to control for more potential confounders than earlier studies.

Differences in the MBRs pose potential limitations. An important example is high numbers of missing values for several variables, including maternal smoking. However, a previous study on hyperemesis and risk of short-term adverse outcomes for offspring, using the Norwegian MBR, included sub-analysis specifically exploring the possible impact of smoking on risks associated with hyperemesis, with no change in the observed associations [5]. Although different ICD-codes were used at different time-points to register hyperemesis, clinically relevant cases are most likely to have been included, regardless of ICD version. Moreover, the prevalence of hyperemesis among controls was relatively low compared to earlier estimates, possibly due to underreporting of mild hyperemesis to the birth- and patient registries, the latter requiring hospital admission. It could also reflect the lack of a general consensus regarding the correct definition of the condition.

Another possible limitation is that information on severity and onset of hyperemesis was unavailable. However, widely used definitions of hyperemesis, including the one applied by the ICD-10, pinpoints time of onset before the end of 22^nd^ week of gestation. Previous research has shown that the majority of women experience hyperemesis during the first and second trimesters [38]. The validity of hyperemesis registration in the MBR has been explored in Norway, but not in Denmark or Sweden. In Norway, investigators reported a sensitivity and specificity of 83.9 % and 96.0 % respectively - satisfactory validity for large-scale epidemiological studies. A risk of misclassification of hyperemesis was also reported, which could result in registration of fewer cases of severe hyperemesis. This may in turn weaken observed associations of exposure and outcome [39].

## Conclusion

We found no association between hyperemesis and the over-all or site-specific risk of cancer in offspring, except for a suggested increase in lymphoma in adolescence and early adulthood. The latter finding may be due to chance and requires validation in other studies.

## Abbreviations

UKCCS: United Kingdom Childhood Cancer Study
MBRs: Medical Birth Registries
NPR: National Patient Registry
RR: Relative risk
CI: Confidence interval
hCG: Human chorionic gonadotropin
HL/NHL: Hodgkin and non-Hodgkin lymphoma
EBV: Epstein Barr virus
